# Single Mathematical Parameter for Evaluation of the Microorganisms’ Growth as the Objective Function in the Optimization by the DOE Techniques

**DOI:** 10.3390/microorganisms8111706

**Published:** 2020-10-31

**Authors:** Maciej Konopacki, Adrian Augustyniak, Bartłomiej Grygorcewicz, Barbara Dołęgowska, Marian Kordas, Rafał Rakoczy

**Affiliations:** 1 Department of Chemical and Process Engineering, Faculty of Chemical Technology and Engineering, West Pomeranian University of Technology in Szczecin, Piastów Avenue 42, 71-065 Szczecin, Poland; adrian.augustyniak@zut.edu.pl (A.A.); mkordas@zut.edu.pl (M.K.); rrakoczy@zut.edu.pl (R.R.); 2 Department of Laboratory Medicine, Chair of Microbiology, Immunology and Laboratory Medicine, Pomeranian Medical University in Szczecin, Powstańców Wielkopolskich Avenue 72, 70-111 Szczecin, Poland; b.grygorcewicz@gmail.com (B.G.); barbara.dolegowska@pum.edu.pl (B.D.); 3 Building Materials and Construction Chemistry, Technische Universität Berlin, Gustav-Meyer Allee 25, 13355 Berlin, Germany

**Keywords:** mathematical description, bacteria cultivation, growth kinetics, optimization process, bacteriology, bacterial physiology

## Abstract

The cultivation of bacteria sets a ground for studying biological processes in many scientific disciplines. The development of the bacterial population is commonly described with three factors that can be used to evaluate culture conditions. However, selecting only one of them for the optimization protocol is rather problematic and may lead to unintended errors. Therefore, we proposed a novel mathematical approach to obtain a single factor that could be used as the objective function to evaluate the whole growth dynamic and support the optimization of the biomass production process. The sigmoidal-shape curve, which is the commonly used function to plot the amount of biomass versus time, was the base for the mathematical analysis. The key process parameters, such as maximal specific growth rate and lag-phase duration were established with the use of mathematical coefficients of the model curve and combined to create the single growth parameter. Moreover, this parameter was used for the exemplary optimization of the cultivation conditions of *Klebsiella pneumoniae* that was cultured to be further used in the production of lytic bacteriophages. The proposed growth parameter was successfully validated and used to calculate the optimal process temperature of the selected bacterial strain. The obtained results indicated that the proposed mathematical approach could be effortlessly adapted for a precise evaluation of growth curves.

## 1. Introduction

**Figure 1:**
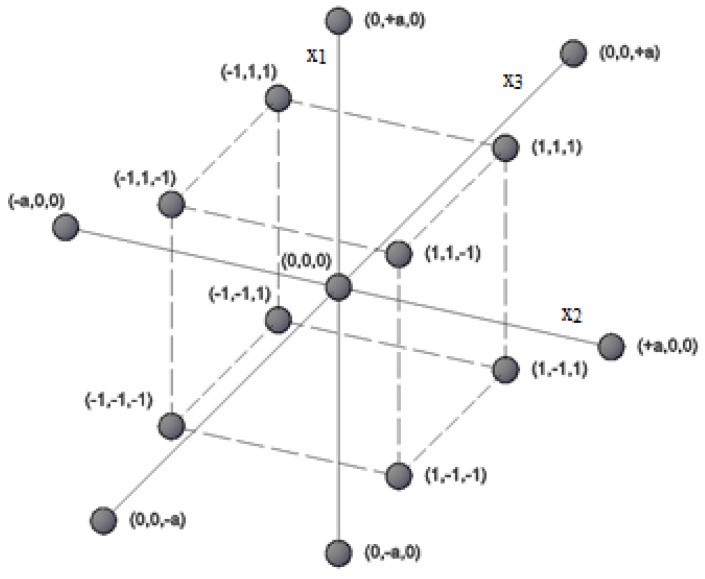
Experimental points given by the central composition design.

The rising demand for biotechnological products as well as for the development of new bioprocesses requires continuous studies on microbial physiology and population growth [1,2]. In recent years, numerous studies on the optimization of cultivation conditions (e.g., medium composition, temperature, pH level) were conducted, which improved the mathematical description of bioprocesses [3,4,5,6,7]. Furthermore, the developments also regarded technological systems where bioreactors were equipped with new types of impellers, electromagnetic coils or ultrasound generators [8,9,10]. All these factors can affect the growth of selected microorganisms, thus the cultivation process should be optimized before every further manipulation. This implies a high number of variables that significantly increase the demand for experiments if one wishes to study every possibility. Design of Experiment (DOE) provides research plans that help with the choice of the experimental points for studied ranges of variables and then the creation of the response surface that describes the analysed region. For example, considering only three variables such as temperature, pH level and impeller speed for five different values from a selected range, a total of 125 experiments need to be conducted in order to create the response surface. Employing one of the DOE designs—central composition plan, the total number of experiments was reduced to 15. Additionally, such a plan assumes also star points +a and -a situated along each axis that allows the estimation of the response surface curvature (where star points are situated in some distance a from the centre point, |a|>1) [11,12,13]. The experimental area described by this design was illustrated in Figure 1.

Based on the points given in Figure 1, a variable input matrix that determines the number of experiments is then created. It should be noticed, that all variables *x*_1_–*x*_3_ presented in Figure 1 and in Table 1 were standardized (all values are illustrating the distance from the middle point 0 at every variable axis). An example of a variable input matrix is presented in Table 1.

The dataset received from such design allows to create a mathematical function and to find its parameters to calculate and optimize the objective. This function can be specified as follows:

$$y(x_{1},x_{2}\ldots x_{n})=p_{0}+\sum_{i=1}^{n}p_{i}x_{i}+\sum_{i=1,j=1}^{n}p_{ij}x_{i}x_{j}$$

where: *p_0,_ p_i_, p_ij_—*function parameters.

Nevertheless, in the optimization protocol, the objective function *y* should be a single variable (making the whole procedure easier, although the multi-objective optimization approach is also possible, even though it is more complicated [14]) such as product or biomass concentration. However, growth process is described not only by the amount of biomass but also by the growth dynamic and to date there is no empirically-validated single parameter that could describe the whole bacteria growth process.

The most common way to illustrate the population growth is by drawing the growth curve that is defined as the function of the number of organisms (or logarithm of it) versus time. Optical density (OD), or colony-forming units (CFU) per millilitre are commonly used as indicators for the number of cells. The typical shape of the growth curve is sigmoid; therefore, three main phases can be marked: lag phase, logarithmic growth and stationary phase. From the perspective of the growth dynamics, each phase could be described by a single parameter. Thus, the suitable mathematical equation describing the growth curve should have at least three coefficients giving the opportunity to calculate all those parameters. The two most popular equations are the Gompertz model and simple logistic function [15,16].

The optimization procedure is crucial to obtain a higher biomass production and should precede the implementation of every bioprocess. For example, many bacteria are cultivated at 37 °C [17], because this value usually sustains the growth of human and animal pathogens such as *Klebsiella pneumoniae* in the diagnostic process [18]. However, this does not mean that the production of microbial biomass would be optimal at this temperature. As opposed to common bacterial models such as *E. coli*, to the best of our knowledge, there are no optimisation studies on the production of *K. pneumoniae* biomass. These bacteria may cause nosocomial infections that may lead to septicaemia and the patient’s death. Isolates that are resistant to antibiotics are particularly hard to combat and therapeutic options are strictly limited. In that case, other solutions have been proposed, including the use of bacteriophages [19]. These viruses can eliminate selected bacteria and may be used to treat critically ill patients; however, they have to be grown on the target bacterium. Therefore, biomass production is essential for this application [20,21,22,23]. The biomass of these pathogens should be also produced to obtain antigens for autovaccines [24]. Furthermore, the biomass of *K. pneumoniae* has been used in the production of R-acetoin [25]. In order to maintain the process in the most efficient way, the cultivation parameters should be first optimized. Nevertheless, using the growth curve to find the optimal growth conditions may be hindered due to the lack of a single evaluation parameter, relating to the whole process which could be defined as the target function for the optimization process. Many researchers choose only one parameter, such as biomass concentration (without taking growth dynamic into account) or specific growth rate that is strictly associated with the logarithmic growth phase and omits the lag time and the maximal biomass concentration that could be obtained in given conditions [26,27].

Therefore, the current study aims to create and verify the mathematical description of the microorganisms’ growth curve that allows the estimation of the growth parameters and that could be used as the objective function in the optimization procedure. In this case, we used *K. pneumoniae* as an example to describe the optimization procedure based on the growth curve observation that was influenced by the cultivation conditions changes. In the current study, we focused to show how the proposed novel parameter works, so in the experimental part we used only temperature changes to affect the bacterial growth. Nevertheless, the proposed method can be used for changes in every condition because all of them will be visible on the growth curve. This paper is also a preliminary study for further assessment of the optimal growth condition of the bacteria host cells for the bacteriophage production process. Furthermore, this research focuses on the creation of a single growth parameter that allows describing the whole process and that creates the opportunity to precisely compare the growth curves obtained in variable conditions.

## 2. Theoretical Background

### Evaluation of Growth Curves

**Figure 2:**
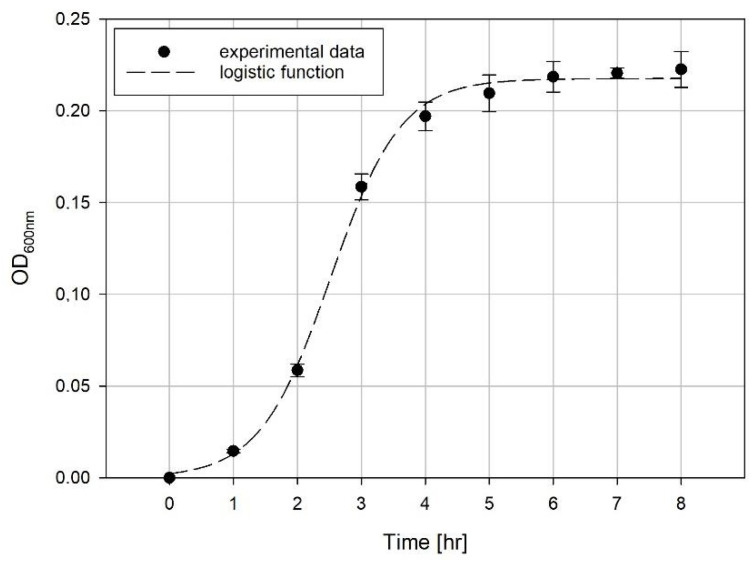
Example of the growth experimental data and model growth curve.

The growth curve is often plotted as optical density (or CFU/mL) versus time. Based on the previous results [17], an example growth curve is presented in Figure 2.

The experimental data can be described mathematically by the means of a logistic curve. In the present work, we decided to employ the following function:

(1) $$y(t)=\frac{a}{1+\exp(b-c\;t)}$$

where: *a* [–], *b* [–], c [hr^−1^]—mathematical function coefficients, *t*—time [hr].

These *a*, *b*, *c* coefficients affect the model growth curve in a specific manner. The impact of each coefficient on the growth curve was described in the Supplementary Materials.

When the growth curve coefficients (given in Equation (1)) are available, a few parameters describing the population growth process can be drawn, including the maximal biomass concentration value—*A* (asymptote), the maximal specific growth rate—$\mu_{\max}$ and the lag time—*λ*. Those three parameters can be specified using the function (1) coefficients as follow:

(2) A=a [-]

(3) $$\mu_{\max}=\frac{a\;c}{4}{\;[\mathrm{hr}}^{-1}]$$

(4) $$\lambda =\frac{b-2}{c}\;[\mathrm{hr}]$$

The detailed derivation of Equations (2)–(4) is presented in the Supplementary Materials.

The three parameters, $A,\;\mu_{\max},\;\lambda$ can be used in the analysis of microbial growth curves [15]. However, a single parameter that describes the whole process could be preferably used, if properly described. For example, a weighted arithmetic mean of these parameters could theoretically serve as such parameter, although the prediction of weight values for each of the three values would be unclear and subjective. For that reason, we decided to propose a different approach based on the analysis of the growth potential by the area under the curve [28], where the mathematical description would result in a novel single parameter that takes into consideration all those three parameters.

Assuming the ideal conditions, the best growth kinetics would be infinitely fast with no lag time, resulting in a maximal possible concentration level that is limited by the asymptote $A_{\max}$, which can be noted as:

(5) $$\mu_{\max}\to \infty ,\;\lambda \to 0,\;A=A_{\max}$$

For such an assumption, the ideal growth curve would be rectangular with a height of $A_{\max}$ and a length of *t*. In that case, the ideal growth potential could be described as:

(6) $$F=A_{\max}\;t$$

**Figure 3:**
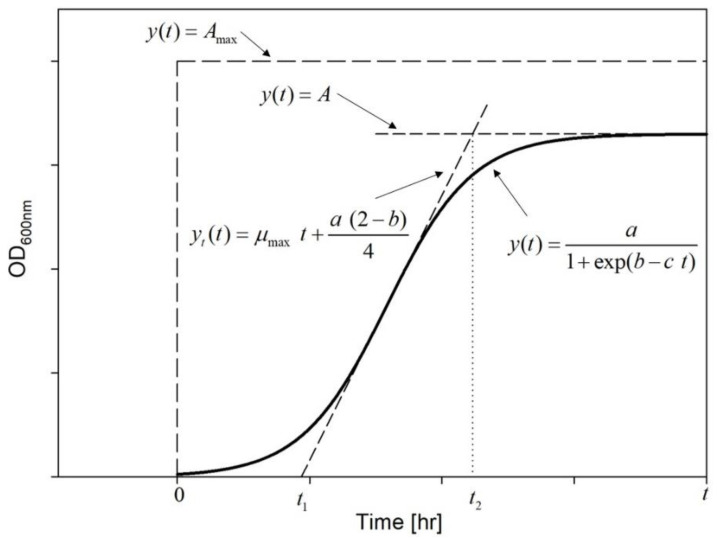
Schematic representation of the area under the growth curve together with the mathematical function describing boundary lines.

In a realistic situation, the growth curve area is mostly limited by the specific growth line $y_{t}\left(t\right)$ and the asymptote A. Both, ideal and realistic, situations are presented in Figure 3.

It should be noticed that the area under the realistic growth curve resembles the trapezoid form. The length of both bases is specified by the time (*t*) and two specific points ($t_{1},\;t_{2}$) given by the interception of the tangent line: first with the *x*-axis and second with the asymptote *A* (see Figure 3). Thus:

(7) $$\begin{array}{l} t_{1}=\lambda \to t_{1}=\frac{b-2}{c}\\ y_{t}\left(t_{2}\right)=A\to t_{2}=\frac{b+2}{c}\; \end{array}$$

The area under the curve for realistic conditions can be defined as:

(8) $$f=A\;\frac{\left(t-t_{1})+(t-t_{2}\right)}{2}$$

The combined form of Equations (7) and (8), is drawn as follows:

(9) $$f=a\;t-\frac{a\;b}{c}$$

The single parameter that we have decided to use in the evaluation of growth curves is the ratio between the area under curves for realistic and ideal conditions:

(10) $$\varphi =\frac{f}{F}$$

After having substituted Equations (6) and (9) to Equation (10), the single growth parameter is received:

(11) $$\varphi =\left(\frac{a}{A_{\max}}\right)\;\left(1-\frac{b}{c\;t}\right)$$

Specifying the maximal growth ratio to be described by $n_{A}=\frac{a}{A_{\max}}$, the final form of the growth parameter is defined as:

(12) $$\varphi =n_{A}\;\left(1-\frac{b}{c\;t}\right)\;[-]$$

This parameter could be used prior to comparing different growth curves obtained in the laboratory experiments, especially for the optimization process. It should be emphasized that the calculated parameter can be utilized only if the experiment time (*t*) is equal for every curve and sufficient large to reach the plateau phase. This allows us to avoid calculation errors. Moreover, the $A_{\max}$ value should be established experimentally at the highest obtained number or assumed from the literature, for a given range of operating conditions. When the maximal growth is the same for all datasets, i.e., $A=A_{\max}$, then the equation describing the growth parameter φ can be simplified to the following formula:

(13) $$\varphi =1-\frac{b}{c\;t}\;[-]$$

Additionally, we performed a visualization of the growth parameter changes by each input parameter (*a*, *b*, *c*) variations by plotting its estimated value in the specified region. The results are presented in the Supplementary Materials.

## 3. Materials and Methods

*Klebsiella pneumoniae* (ATCC^®^ BAA-1706^™^) was used in laboratory experiments. Bacteria were kept frozen in Trypticase Soy Broth medium (TSB) with 10% (*v/v*) glycerol.

Refrozen cultures were streaked to Trypticase Soy Agar (TSA) medium and incubated at 37 °C for 24 h. Afterwards, a colony was transferred to 30 mL of fresh TSB medium and incubated overnight (14–16 h) at 37 °C. In the next step, 300 mL of TSB at the test temperature was inoculated in ratio 1:100 and evenly dispensed to the 15 mL Falcon tubes (10 mL of inoculum to each tube). At this time, 8 samples (100 µL each) were taken from the inoculum and medium and their optical density (OD, at λ = 600 nm) was measured on BioTek Synergy H1 (Winooski, VT, USA) spectrophotometer. The experiments were continued for 10 h to achieve the plateau phase. One tube was taken every hour and 8 samples (100 µL each) were subjected to OD measurements. Experiments were led at six selected temperatures, including 25 °C, 27.3 °C, 33 °C, 37 °C, 38.7 °C and 41 °C. Furthermore, the metabolic activity of cells was controlled in MTT and resazurin assays, as described elsewhere [29,30]. In order to perform all tests for each sample, the time selected for the incubation of biochemical tests was 20 min. In the case of MTT, cells were disrupted with DMSO (99.99%, 100 µL per sample) and further incubated for 15 min.

## 4. Results and Discussion

**Figure 4:**
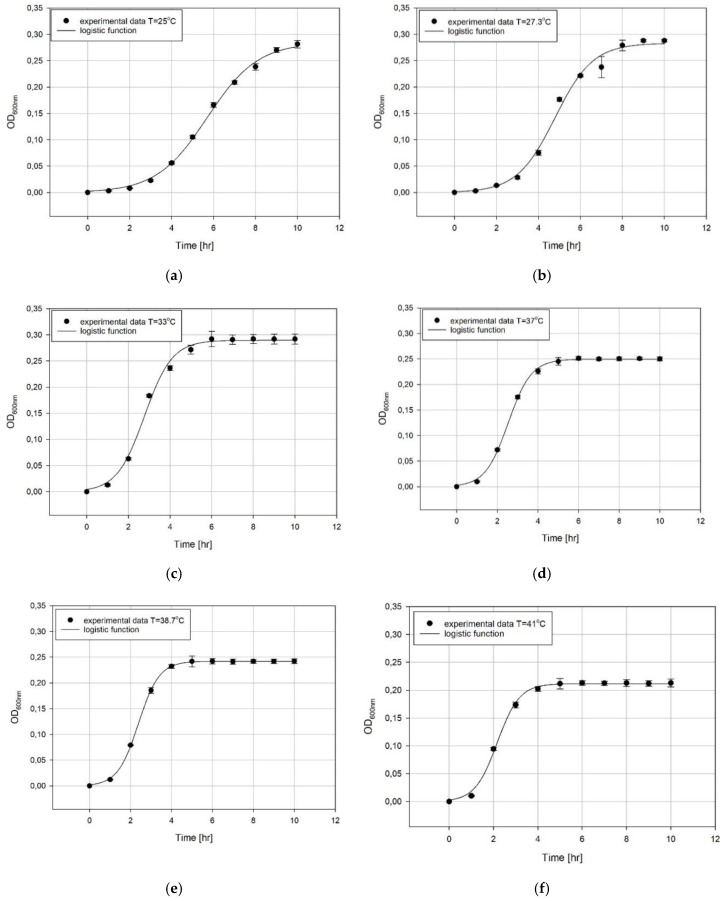
The growth of *K. pneumoniae* in various temperatures: (**a**) T = 25 °C, (**b**) T = 27.3 °C, (**c**) T = 33 °C, (**d**) T = 37 °C, (**e**) T = 38.7 °C, (**f**) T = 41 °C.

The experimental data of *K. pneumoniae* cultures in the tested range of temperatures are presented in Figure 4. The cultures were led until they all reached the plateau phase. As a result of the slower growth at 25 °C, the curves were prolonged up to the 10th hour.

The obtained dataset was described by the logistic function Equation (1) using the Statistica 13 (Statsoft, Kraków, Poland) software and the least square error method. In result, the function coefficients were obtained with a very good model adjustment. All obtained values are presented in Table 2.

**Figure 5:**
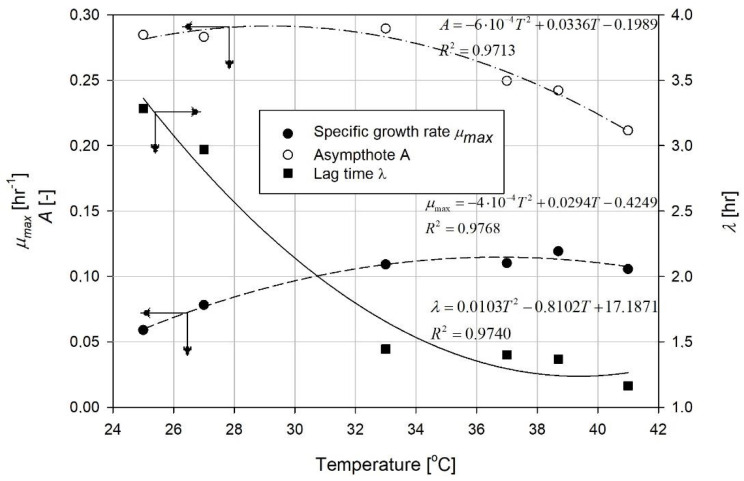
Changes of growth parameters with the process temperature. The approximations were conducted by the method of least squares.

Maximal growth, maximum specific growth rate and lag time were calculated with the use of Equations (2)–(4), respectively. The results are presented in Figure 5.

The maximum specific growth rate (Figure 5, filled circles) increased with the temperature up to 33 °C. Afterwards, it changed slightly, fluctuating around 0.11 hr^−1^. The lag time (Figure 5, filled squares) strongly depended on the temperature, especially in the lower region (25–33 °C). Generally, the rise in temperature was followed by a drop in the lag time. Above 33 °C, this effect was slower but also measurable. The maximum growth concentration specified by asymptote was also affected by temperature (Figure 5, empty circles). The final OD reached in the cultures was bigger for the lower range of temperatures (25–33 °C). The further rise in the temperature resulted in a strong decrease in the value of the asymptote.

**Figure 6:**
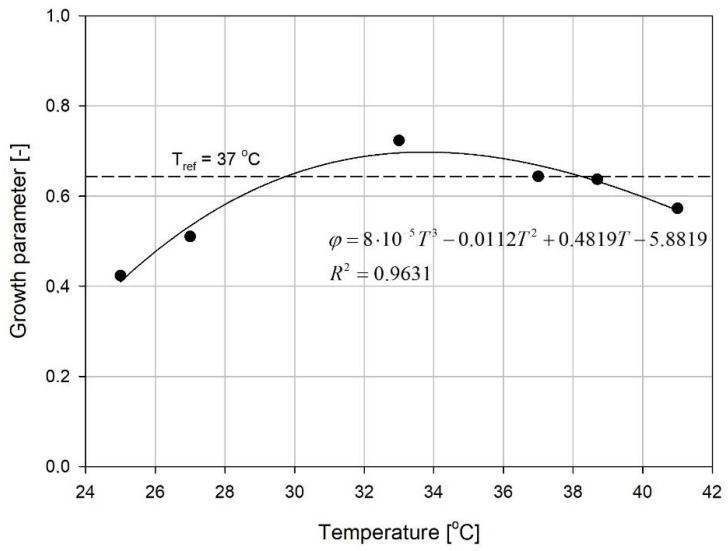
Changes in the growth parameter φ of the process temperature. The approximations were conducted by means of the method of least squares.

Calculating the optimal temperature with the use of 3 parameters is rather complicated thus, the growth parameter φ was calculated according to Equation (13). These results are presented in Figure 6.

The calculated values of growth parameters (Figure 6) presented the region of optimal temperature around 33–34 °C. The population growth was improved by 12% in comparison to the result obtained at 37 °C. Thus, 37 °C has not been the optimal temperature for the cultivation of bacteria, even though it is commonly used in the research on *K. pneumoniae* and other representatives of this genus [2,25]. Therefore, the effective biomass production could be performed at lower temperatures that would reduce the energy (heat) consumption, thus the cost of the process [1]. Our analysis supports the notion that it is crucial to experimentally find the optimal conditions before heading to the production process. A well-fitted curve to growth parameter versus temperature plot would give in that case the precise optimum for the process temperature (in the studied case its 33.8 °C). As shown above, the experimental results confirmed that the obtained φ value was higher at this range of the temperature than at the commonly used 37 °C [31]. Interestingly, it has been suggested in the literature that the best results in biochemical testing of clinical strains of *K. pneumoniae* are obtained at 33–34 °C [18].

## 5. Conclusions

Several parameters, such as maximum specific growth rate or the lag time can describe the growth of bacterial population. Nevertheless, we have shown that the proposed single parameter could be a convenient tool to evaluate the growth during the optimization process. The fitting of the logistic function to the growth curve was relatively simple and produced the adjusted coefficients with very high accuracy. The known values of function coefficients allow the rapid evaluation of the curve with the use of the growth parameter that makes this approach very straightforward and reliable. The experimental data have validated the model showing that the optimal temperature for the cultivation of *K. pneumoniae* is 33.8 °C. The reasoning behind the proposed single parameter has the potential to be further used also with other bacteria than *K. pneumoniae*. The proposed approach can be applied in the optimization of the biomass production for biotechnological processes, including the production of phage-based solutions, or lysates for autovaccines.

## Figures and Tables

**Table 1:** An example of a variable input matrix.

Experiment	1	2	3	4	5	6	7	8	9	10	11	12	13	14	15
*x*_1_	1	−1	1	−1	1	−1	1	−1	−a	+a	0	0	0	0	0
*x*_2_	1	1	−1	−1	1	1	-1	−1	0	0	−a	+a	0	0	0
*x*_3_	1	1	1	1	−1	−1	−1	−1	0	0	0	0	−a	+a	0
*y*	*y*_1_	*y*_2_	*y*_3_	*y*_4_	*y*_5_	*y*_6_	*y*_7_	*y*_8_	*y*_9_	*y*_10_	*y*_11_	*y*_12_	*y*_13_	*y*_14_	*y*_15_

*x*_1_–*x*_3_—standardized input variables (such as temperature, etc.); *y*—the objective function.

**Table 2:** Growth curve model function coefficients in selected temperatures.

Temperature [°C]	Function Coefficients			*R* ^2^
	*a*	*b*	*c*	
25	0.2846	4.7225	0.8289	0.9981
27.3	0.2831	5.2786	1.1033	0.9929
33	0.2895	4.1777	1.5073	0.9959
37	0.2495	1.7655	1.7655	0.9991
38.7	0.2421	4.6926	1.9688	0.9998
41	0.2116	4.3208	1.9952	0.9978

## Supplementary Materials

The following are available online at https://www.mdpi.com/2076-2607/8/11/1706/s1, Figure S1. Impact of model growth curve coefficients: (a) a = var, b, c = const (b = 3, c = 0.6); (b) b = var, a, c = const (a = 0.1, c = 0.6); (c) c = var, a,b = const (a = 0.1, b = 3), Figure S2. The influence of the model function coefficients on the growth parameter (φ) in the constant process time (16 h): (a) a-value (b = 3, c = 0.6), (b) b-value (a = 0.1, c = 0.6), (c) c-value (a = 0.1, b = 3), Figure S3. The influence of b and c coefficients on the growth parameter in the constant process time (16 h) and with A = A_max_.
