# Supplementary material for: Single Mathematical Parameter for Evaluation of the Microorganisms’ Growth as the Objective Function in the Optimization by the DOE Techniques
Source: Microorganisms. 2020 Oct 31;8(11):1706. doi: 10.3390/microorganisms8111706 (PMC7692173; doi:10.3390/microorganisms8111706)
Supplement: Supplementary file 1 [file microorganisms-08-01706-s001.pdf]

Supplementary materials

Impact of logistic function coefficients on the growth curve

Fig. S1 depicts the impact of each coefficient on curve behaviour:

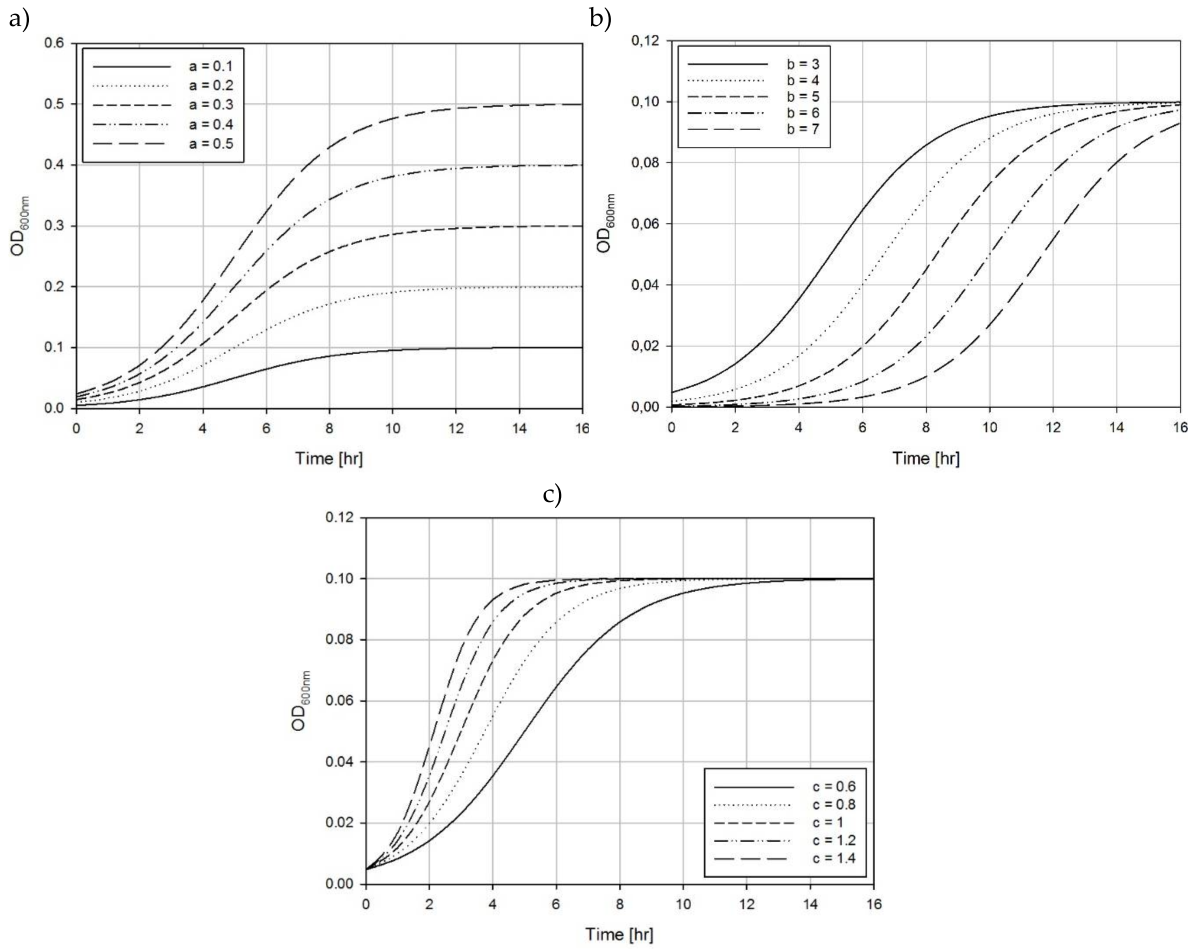

Figure S1. Impact of model growth curve coefficients: a) $a = \text{var},\ b,c = const$ (b=3, c=0.6); b) $b = \text{var},\ a,c = const$ (a=0.1, c=0.6); c) $c = \text{var},\ a,b = const$ (a=0.1, b=3).

Fig. S1a shows that changes in coefficient *a* affect the maximal optical density that can be achieved. Higher *a* value proportionally results in higher OD in the stationary phase. Moreover, the specific growth rate in the logarithmic phase is also changed proportionally with the increase of parameter *a*. Coefficient *b* (Fig. S1b) plays a role only in the duration of the lag phase. Lower *b* value leads to a shorter lag. The last coefficient *c* primarily affects the growth rate in the logarithmic phase (the bigger *c* value the higher specific growth rate), but also has an impact on the lag phase (higher *c* value results in a shortened lag phase). Taking all these parameters into consideration, the growth process is more efficient (with high growth rate and final concentration) when the values of coefficients *a* and *c* are high, and the value of *b* is low.

Derivation of maximum specific growth and lag time

The maximum specific growth rate is given by the slope of the tangent line in the growth curve inflection point t_i. The inflection point can be found when the second-order derivative is equal to zero:

$$t = t_i \rightarrow \frac{d^2 y}{dy^2} = 0 \tag{S1}$$

Using eq. 1, the first and second-order derivatives can be obtained:

$$\frac{dy}{dt} = \frac{a\ c\ \exp(b - c\ t)}{\left[\exp(b - c\ t) + 1\right]^2} \tag{S2}$$

$$\frac{d^2 y}{dy^2} = \frac{2\ a\ c^2\ \exp(2b - 2c\ t)}{\left[\exp(b - c\ t) + 1\right]^3} - \frac{a\ c^2\ \exp(b - c\ t)}{\left[\exp\left(b - c\ t\right) + 1\right]^2} \tag{S3}$$

Simplified combination of eq. S1 and S3 will result in:

$$\begin{aligned} &\frac{2\ a\ c^2\ \exp(2b - 2c\ t_i)}{\left[\exp(b - c\ t_i) + 1\right]^3} - \frac{a\ c^2\ \exp(b - c\ t_i)}{\left[\exp\left(b - c\ t_i\right) + 1\right]^2} = 0 \rightarrow \\ &\rightarrow \exp\left(b - c\ t_i\right) - 1 = 0 \end{aligned} \tag{S4}$$

Finally, solving equation S4 allows to obtain the inflection point:

$$t_i = \frac{b}{c} \tag{S5}$$

The inflection point can be used to estimate the maximum specific growth rate by calculating the first-order derivative at this point:

$$\frac{dy}{dt}_{|t=t_i} = \mu_{\max} \tag{S6}$$

Afterwards, equations S2 and S5 are substituted into equation S6 that result in receiving the following formula:

$$\frac{a\ c\ \exp\left(b - c\ \frac{b}{c}\right)}{\left[\exp\left(b - c\ \frac{b}{c}\right) + 1\right]^2} = \mu_{\max} \tag{S7}$$

Once simplified, the final form of the maximum specific growth rate is defined as follows:

$$\mu_{\max} = \frac{a\ c}{4}\ [\mathrm{hr}^{-1}] \tag{S8}$$

The tangent line $y_t(t)$ at the inflection point can be described by the equation:

$$y_t(t) = \mu_{\max}\ t + b_t \tag{S9}$$

where: b_t – y-intercept coefficient of the tangent line [–]

The coefficient b_t can be found, if the coordinates of the inflection point are inserted into eq. S9, which is also part of the growth curve (so $y_t\left(t_i\right) = y\left(t_i\right)$):

$$y_t\left(t_i\right) = \mu_{\max}\ t_i + b_t \rightarrow b_t = y\left(t_i\right) - \mu_{\max}\ t_i \tag{S10}$$

Applying equations 1, S5, S8, and S10 together results in the following formula:

$$\begin{aligned} b_t &= \frac{a}{1+\exp\left(b - c\ \frac{b}{c}\right)} - \frac{a\ c}{4}\ \frac{b}{c} \\ b_t &= \frac{a\ (2-b)}{4} \end{aligned} \tag{S11}$$

Thus, the final form of the tangent line is expressed as follows:

$$y_t(t) = \mu_{\max}\ t + \frac{a\ (2-b)}{4} \tag{S12}$$

The lag time, i.e. the period until the cells reach the logarithmic growth phase, can be described as the x-intercept of the specific growth rate tangent line, so:

$$y_t\left(t\right)_{|t=\lambda} = 0 \tag{S13}$$

By combining equations S8, S12, and S13, and solving them we obtained the lag time parameter:

$$\lambda = \frac{b-2}{c}\ \text{[hr]} \tag{S14}$$

Impact of function coefficients on the single growth parameter

The impact of the function (1) coefficients on the growth parameter (eq.12) is presented in Fig. S2

a) b)

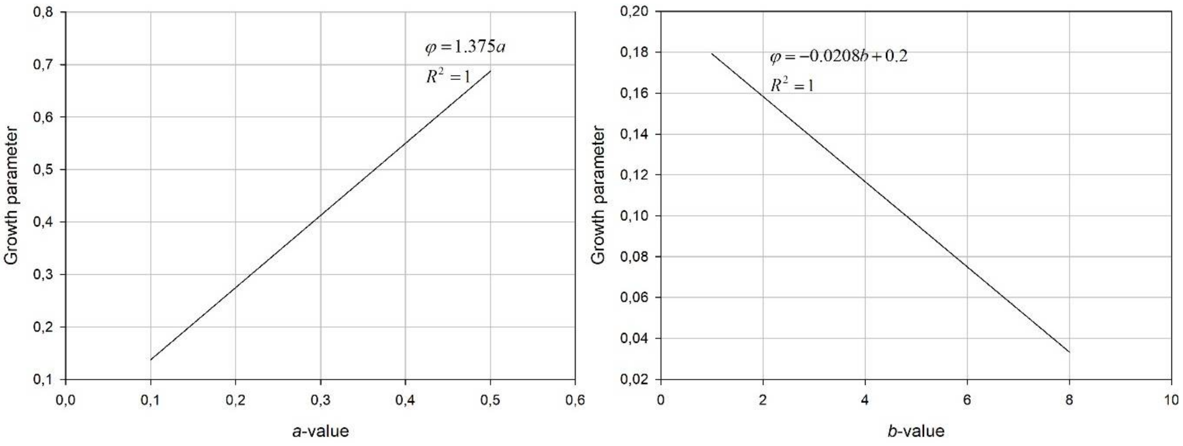

c)

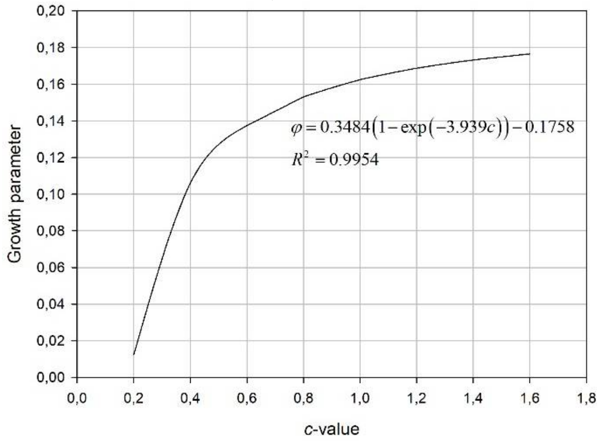

Figure S2. The influence of the model function coefficients on the growth parameter (φ) in the constant process time (16h): a) *a*-value (*b*=3, *c*=0.6), b) b-value (*a*=0.1, *c*=0.6), c) *c*-value (*a*=0.1, *b*=3).

The results show a good agreement between the growth curve behaviour and the growth parameter φ. The changes in *a*-value and *b*-value cause proportional alteration in the growth curve (see Fig. S1 a and b). The same can be observed for the growth parameter φ (Fig. S2 a and b). An increase in *c*-value up to 0.5 causes a sharp rise in the growth parameter, whereas under this point the relation continues more slowly. The same correlation can be observed for growth curves (Fig. S1 c), which proves the high sensitivity of the proposed single parameter. In the case of the same maximal growth in every experiment ($A = A_{\max} = const$), the growth parameter depends only on coefficients *b* and *c*. The relationship between φ, *b*, and *c* parameters are presented on the surface plot in Fig. S3.

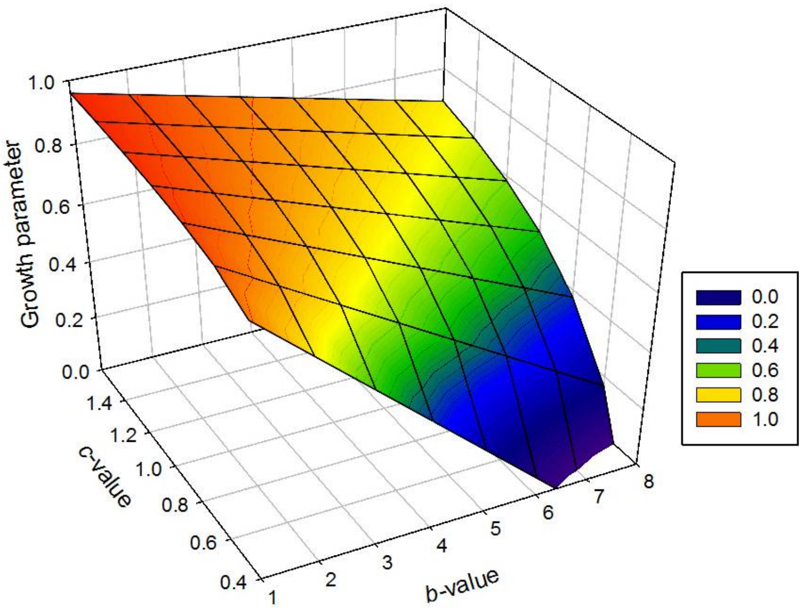

Figure S3. The influence of b and c coefficients on the growth parameter in the constant process time (16h) and with $A = A_{max}$.

Furthermore, these relationships also strongly correlate with the growth curve changes shown in Fig. 2. The higher *b*-values are associated with the lag phase and cause a sharp fall in the value of the growth parameter even to 0 (e.g. *b*=6.4, *c*=0.4, thus λ=16h). The coefficient *c* has a higher impact than the region of low values and smaller for higher values that confirms the previous findings.
